# Ct-Guided Pancreatic Percutaneous Fine-needle Biopsy in Differential Diagnosis Between Pancreatic Cancer and Chronic Pancreatitis

**DOI:** 10.1155/1989/84039

**Published:** 1989

**Authors:** Michele Carlucci, Alessandro Zerbi, Danilo Parolini, Sandro Sironi, Angelo Vanzulli, Carlo Staudacher, Agostino Faravelli, Paola Garancini, Alessandro del Maschio, Valerio di Carlo

**Affiliations:** 1 Cattedre di Patologia Speciale Chirurgica Istituto Scientifico H.S. Raffaele Università di Milano Italy; 2 Cattedre di Radiologia Istituto Scientifico H.S. Raffaele Università di Milano Italy; 3 Cattedre di Anatomia Patologica Istituto Scientifico H.S. Raffaele Università di Milano Italy; 4 Cattedre di Statistica Medica Istituto Scientifico H.S. Raffaele Università di Milano Italy; 5 Cattedra Patologia Chirurgica Istituto Scientifico H.S. Raffael Universita' di Milano Via Olgettina 60 Milano 20132 Italy

## Abstract

Differential diagnosis between pancreatic cancer and chronic pancreatitis is still difficult to establish. In 63 patients with suspected pancreatic neoplasm we performed: serum CA 19-9 assessment, abdominal ultrasound, CT scan and CT-guided pancreatic percutaneous fine-needle biopsy. The conclusive diagnosis was pancreatic cancer in 40 patients and chronic pancreatitis in 23 patients. With regard to the differential diagnosis, sensitivity and specificity were respectively 80% and 78%  for serum CA 19-9, 75%  and 65%  for abdominal US, 85% and 70%  for CT scan, 00% and 87%  for percutaneous fine-needle biopsy. We conclude that CT-guided percutaneous fine-needle biopsy is the most reliable method for differential diagnosis between pancreatic cancer and chronic pancreatitis.


*HPB Surgery*, 1989, Vol. 1, pp. 309–317
Reprints available directly from the publisher
Photocopying permitted by license only
© 1989 Harwood Academic Publishers GmbH
Printed in Great Britain

### CT-GUIDED PANCREATIC PERCUTANEOUS FINE-NEEDLE BIOPSY IN DIFFERENTIAL DIAGNOSIS BETWEEN PANCREATIC CANCER AND CHRONIC PANCREATITIS

MICHELE CARLUCCI* ALESSANDRO ZERBI, DANILO PAROLINI, SANDRO SIRONI°, ANGELO VANZULLI°, CARLO STAUDACHER, AGOSTINO FARAVELLI+, PAOLA GARANCINI**, ALESSANDRO DEL MASCHIO° and VALERIO DI CARLO*

*Cattedre di Patologia Speciale Chirurgica, Radiologia°, Anatomia Patologica+* e Statistica Medica** – *Istituto Scientifico H.S. Raffaele- Università di Milano-Italy*

*(Received 29 June, 1988)*

Differential diagnosis between pancreatic cancer and chronic pancreatitis is still difficult to establish. In 63 patients with suspected pancreatic neoplasm we performed: serum CA 19-9 assessment, abdominal ultrasound, CT scan and CT-guided pancreatic percutaneous fine-needle biopsy. The conclusive diagnosis was pancreatic cancer in 40 patients and chronic pancreatitis in 23 patients. With regard to the differential diagnosis, sensitivity and specificity were respectively 80% and 78% for serum CA 19-9, 75% and 65% for abdominal US, 85% and 70% for CT scan,[1] 00% and 87% for percutaneous fine-needle biopsy. We conclude that CT-guided percutaneous fine-needle biopsy is the most reliable method for differential diagnosis between pancreatic cancer and chronic pancreatitis.

KEY WORDS: Pancreatic cancer, chronic pancreatitis, CT scan, fine-needle biopsy.

### INTRODUCTION

Differential diagnosis between pancreatic cancer and chronic pancreatitis is still difficult to establish today despite the increasingly widespread use of tumors markers[1,2,3] and the recent progress made in the field of instrumental diagnosis.

Large scale use of ultrasonography (US) and computerized tomography (CT) has actually worsened the situation by bringing to light more cases of focal pancreatic lesions which are more or less asymptomatic[4]; it is not easy to establish if these lesions are due to inflammation or a neoplasm. Pancreatic percutaneous fine-needle biopsy has recently been utilized more in the diagnostic field[5,6,7,8] due to improvements in the needles and the type of guide used[9].

The aim of this study is to evaluate the contribution made by CT guided pancreatic percutaneous fine-needle biopsy to differentiate between pancreatic cancer and chronic pancreatitis.

* Correspondence to Michele Carlucci, M.D. – Cattedra Patologia Chirurgica – Istituto Scientifico H.S. Raffaele – Universita' di Milano – Via Olgettina 60 – 20132 Milano – ITALY.

Results obtained with fine-needle biopsy were compared to those obtained with CA 19-9 assay and with non invasive techniques (US, CT).

### MATERIALS AND METHODS

Between December '84 and March '88, 106 patients were admitted to our hospital for suspected pancreatic neoplasm. 43 patients were excluded from this study as they were discovered to have non pancreatic pathology. The remaining 63 patients, 37 male and 26 female, average age 61 years, range 32–83 years, with a final diagnosis of pancreatic cancer or chronic pancreatitis were included in this study.

24 patients were jaundiced (19 with pancreatic cancer and 5 with chronic pancreatitis). In all patients a focal pancreatic lesion was observed; the average diameter of the punctured lesion was 4,4 cm, range 2–8 cm; the lesion was located in 39 patients in the head of the pancreas, in 18 in the body and in the remaining 6 in the tail.

All the patients were subjected to the following diagnostic procedures:
-serum CA 19–9 assay by an immunoradiometric technique (Centocor – Sorin Biomedica, Saluggia VC, Italy); a cut-off value of 40 U/ml was applied[10].
-abdominal US, using 3,5 and 5 MHz probes.
-abdominal CT carried out with third generation model after rapid intravenous injection of the contrast medium; 10 mm axial scans were done, and 5 mm scans in the areas of most interest.

The images obtained from these procedures were examined by a staff radiologist without knowledge of the cases, and classified either as positive for neoplasm, negative for neoplasm or non-diagnostic.

During the CT scanning, pancreatic percutaneous fine-needle biopsy was performed. After the exact localization of the lesion and the decision about the best area for cytological examination, the exact spot of the scan was marked on the skin with the luminous collimator of the CT gantry and subsequently the needle was inserted, after local anaesthetization. Most specimens were taken with Chiba or Franzen 22 G needles. The sample was drawn percutaneously and anteriorly. The obtained smears were immediately fixed in an alcohol-ether solution, rapidly stained with haematoxylin-eosin and immediately examined by the pathologist. When the material was inadequate, another sample was taken using larger needles (20 G). An average of 1,5 specimens was taken, range 1-3. The final results were expressed as either positive for malignant tumor cells (MTC), negative for MTC or inadequate material. After fine-needle biopsy all the patients were given a CT scan to ensure that there were no blood clots. In the following days the patients were given a clinical examination and analyses were carried out (determination of amylase in the serum and urine) to exclude the onset of pancreatitis.

Of the 63 patients 35 (55%) underwent surgery, during which we looked for lesions which could possibly be due to percutaneous fine-needle aspiration. A conclusive diagnosis was made in these 35 patients by histological examination of the surgical specimen (10 cases) or by cytological examination of the intraoperative pancreatic fine-needle biopsy (25 cases). In all cases this biopsy confirmed the pre-operative CT-guided pancreatic fine-needle biopsy; an average of 3 specimens was taken, range 1-6. In the remaining 28 patients (45%) who did not have surgery, the conclusive diagnosis was made by means of the aforesaid diagnostic procedures and, in case of chronic pancreatitis, also by means of exocrine pancreatic function test

(Pancreolauryl-test)[11]. In all patients the conclusive diagnosis was confirmed by the clinical follow-up, which included monthly records of symptoms and physical examination; routine laboratory tests, CA 19-9 assessment, abdominal US and chest X-ray were performed every three months. A follow-up period of at least 6 months was required to be included in the study.

In order to assess the accuracy of the considered diagnostic procedures we estimated their own sensitivity, specificity and predictive value for cancer: sensitivity represents the probability that a subject with cancer will give a positive result for cancer with the test; specificity represents the probability that a subject without cancer (i.e. chronic pancreatitis) will give a negative result for cancer with the test and predictive value is the probability that a positive subject will be actually suffering from cancer. Finally, 95% confidence limits were calculated for sensitivity, specificity and predictive value[12].

### RESULTS

Of the 63 patients in the study, 40 (63%) were shown to be affected by pancreatic adenocarcinoma and 23 (37%) by chronic pancreatitis on the basis of the conclusive data.

Serum CA 19-9 was over 40 U/ml in 32 patients (80%) with pancreatic carcinoma and in 5 patients (22%) with chronic pancreatitis.

Abdominal US was positive for neoplasm in 30 patients (75%) with pancreatic cancer and in 2 patients (9%) with chronic pancreatitis. It was negative for neoplasm in 15 patients (65%) with chronic pancreatitis and in none of the patients with pancreatic cancer. It was non-diagnostic in 10 patients (25%) with pancreatic cancer and in 6 patients (26%) with chronic pancreatitis.

Abdominal CT was positive for neoplasm in 34 patients (85%) with pancreatic carcinoma and in 1 patient (4%) with chronic pancreatitis. It was negative for neoplasm in 3 patients (7%) with pancreatic cancer and in 16 patients (70%) with chronic pancreatitis. It was non-diagnostic in 6 patients (26%) with chronic pancreatitis and in 3 patients (7%) with pancreatic cancer.

CT- guided pancreatic percutaneous fine-needle biopsy was positive for MTC in all of the 40 patients with pancreatic cancer (100%) and in none of the patients with chronic pancreatitis. It was negative for MTC in 20 patients with chronic pancreatitis (87%). The material was unsuitable for assessment in the remaining 3 patients with chronic pancreatitis (13%) (Table 1).

**Table 1** Results of serum C A 19-9 assessment, abdominal ultrasound (U.S.), abdominal C.T. scan (C.T.), C.T.-guided fine-needle biopsy (biopsy) in 40 patients with pancreatic cancer and 23 patients with chronic pancreatitis.

| | *Pancreatic cancer 40 pts* | | | *Chronic pancreatitis 23 pts* | | |
|---|---|---|---|---|---|---|
| | Pos. (*) | Neg. | N.D. | Pos. | Neg(**) | N.D. |
| CA 19-9 | 80% | 20% | – | 22% | 78% | – |
| U.S. | 75% | 0% | 25% | 9% | 65% | 26% |
| C.T. | 85% | 7% | 7% | 4% | 70% | 26% |
| BIOPSY | 100% | 0% | 0% | 0% | 87% | 13% |

Pos. = positive for neoplasm, Neg. = negative for neoplasm, N.D. = non diagnostic.
(*) = Sensitivity (**) = Specificity

In regard of the differential diagnosis between pancreatic cancer and chronic pancreatitis, sensitivity, specificity and predictive value were respectively 80% 78% and 86% for serum CA 19-9, 75% 65% and 94% for abdominal US, 85% 70% and 97% for CT scan, 100% 91% and 100% for percutaneous fine-needle biopsy.

95% confidence limits for the four diagnostic procedures were respectively, in case of sensitivity 64%–90%, 58%–87%, 69%–94%, 94%–100%, in case of specificity 56%–92%, 35%–76%, 40%–79%, 65%–97% and, in case of predictive value 70%–95%, 78%–99%, 83%–100%, 89%–100% (Figure 1).

In none of the patients did the CT scans done after fine-needle biopsy show evidence of peri-pancreatic blood clots. There was no significant change in the level of serum or urinary amylase, nor was there clinical evidence of pancreatitis. Lesions attributable to percutaneous fine-needle biopsy were never found on surgery, except for one case where a haemorrhagic collection was present in the greater omentum.

### DISCUSSION

The results of our study confirm that it is still difficult to obtain an accurate differential diagnosis between pancreatic cancer and chronic pancreatitis. In our experience, as already pointed out by Bodner[13], it is easier to make a diagnosis of malignancy on cytological material than on frozen sections.

We compared the accuracy of serum CA 19-9 assessment, abdominal US, CT scanning and CT guided pancreatic percutaneous fine-needle biopsy. Serum CA 19-9 proved to be a sensitive and specific tumour marker for pancreatic cancer[2,3]. However, its diagnostic reliability is limited by the number of false negative results and false positive results in the case of obstructive jaundice[3,14] and active pancreatitis[2]. The usefulness of serum CA 19-9 assay is more in indicating the prognosis and as an early marker of relapse in patients who had undergone surgery for pancreatic cancer[15].

There are serious technical limitations with abdominal US, mainly due to the difficulty in visualising the pancreas if a great amount of intestinal gas is present[16]. These limitations were responsible for 10 non-diagnostic cases (25%); sensitivity (75%) and specificity (65%) are similar to those found in the literature[16,17]. In our experience US would therefore seem to have a limited role in differential diagnosis between chronic pancreatitis and pancreatic cancer.

The limitation of CT scanning is its frequent inability to distinguish the type of lesion. However our results cannot prove any statistically significant difference among the accuracy of serum CA 19-9 assessment, abdominal US and CT scanning (Figure 1). On the other hand the sensitivity of CT guided pancreatic percutaneous fine-needle biopsy appeared statistically higher (94%–100%) as compared with the three previous procedures.

Our experience with pancreatic percutaneous fine-needle biopsy proved very favourable. The procedure is simple and quick; an average of 1,5 aspirations for each case were taken, which gradually decreased with the experience of the operator. The CT guide was able to clearly indicate the blood vessels, the best point for needle insertion into the lesion and it was unhindered by intestinal gas. This method proved very reliable in differential diagnosis between pancreatic cancer and chronic pancreatitis. As far as the specificity is concerned, its results were influenced by 3 inadequate specimens in 3 cases of chronic pancreatitis. The fibrosis often present in cases of chronic pancreatitis is in fact a limiting factor in obtaining material for a

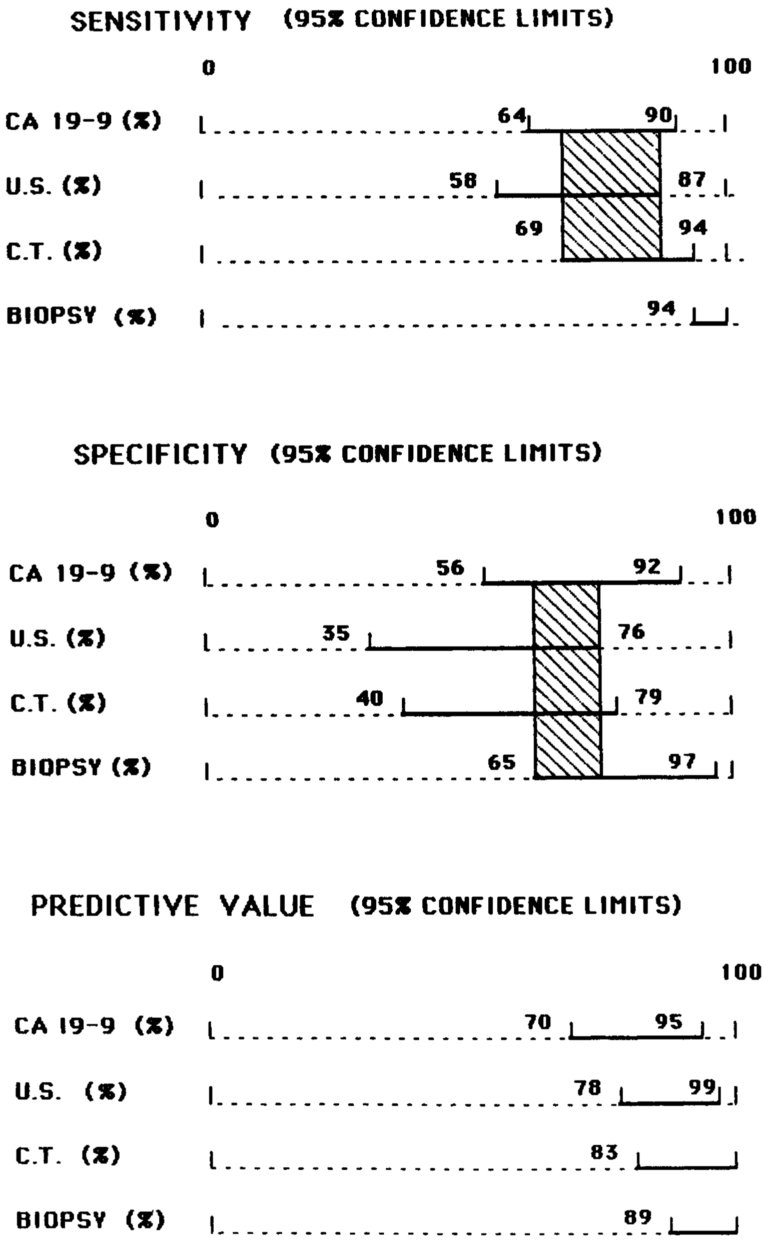

**Figure 1** 95% confidence limits for serum CA 19-9 assessment (CA 19-9), abdominal ultrasound (U.S.), abdominal C.T. scan (C.T.) and pancreatic percutaneous fine-needle biopsy (biopsy), calculated for sensitivity, specificity and predictive value.

cytological diagnosis. On the other hand, the finding of numerous inadequate specimens in different fine-needle biopsies is in our experience suggestive of chronic pancreatitis.

The percutaneous biopsy procedure we used was without immediate complications such as haemorrhage or pancreatitis. Only in one case there was a mild omental haemorragic collection, of no clinical significance. This method would seem to be safe and simple, and the risk of spreading neoplastic cells as reported in the literature would appear even more remote with increasing expertise in this technique[8,18].

In conclusion, CT-guided pancreatic percutaneous fine-needle biopsy was found to be the most reliable method, in terms of sensitivity, to differentiate pancreatic cancer by chronic pancreatitis, superior to tumor markers and the non-invasive techniques such as US and CT scanning. With this method diagnostic explorative laparotomy can be avoided in selected cases.

Finally, the best diagnostic procedure for a patient with suspected pancreatic cancer should include initially serum CA 19-9 assessment and abdominal US; if these procedures are positive or, even if they are negative, and there is a persistent clinical suspicion of cancer, we think that CT scanning associated with pancreatic percutaneous fine-needle biopsy is necessary.

### *References*

1. Del Villano, B.C., Brennan, S., Brock, P *et al* (1983) Radioimmunometric assay for a monoclonal antibody-defined tumor marker. *Clin. Chem.*, **29,** 549–552
2. Malesci, A., Tommasini, M.A., Bonato, C. *et al* (1987) Determination of CA 19-9 antigen in serum and pancreatic juice for differential diagnosis of pancreatic adenocarcinoma from chronic pancreatitis. *Gastroenterology,* **92,** 61–67
3. Malesci, A., Tommasini, M.A., Bocchia, P. *et al* (1984) Differential diagnosis of pancreatic cancer and chronic pancreatitis by a monoclonal antibody detecting a new cancer associated antigen (CA 19-9). *Ricerca Clin. Lab.*, **14,** 303–306
4. Hessel, S.J. *et al* (1982) A prospective evaluation of Computed Tomography and Ultrasound of the pancreas. *Radiology,* **143,** 129–133
5. Smith, E.H., Bartrum, R.J., Chang, Y.C. *et al*(1975) Percutaneous aspiration biopsy of the pancreas under ultrasonic guidance. *N. Eng. J. Med.*, **292,** 825–828
6. Gudjonsson, B., Spiro, H.M. (1978) Biopsy techniques in the diagnosis of pancreatic cancer. *Gastroenterology,* **75,** 726–728
7. Ferrucci, J.T. jr., Wittenberg, J., Mueller, P.R. *et al* (1980) Diagnosis of abdominal malignancy by radiologic fine-needle aspiration biopsy. *AJR,* **134,** 323–330
8. Hancke, S. *et al* (1975) Ultrasonically guided percutaneous fine-needle biopsy of the pancreas. *Surgery, Gynecology and Obstetrics,* **140,** 361–364
9. Ferrucci, T.J. jr. *et al* (1985) Interventional Radiology of the abdomen, Baltimore: *Williams and Wilkins*
10. Ritts, R.E. jr., Del Villano, B.C., GO, V.L.W., Herzerman, R.B., Klug, T.L., Zurawski, V.R. jr. (1984) Initial clinical evaluation of an immunoradiometric assay for CA 19-9 using the NCI serum bank. *Int. J. Cancer,* **33,** 339–345
11. Ventrucci, M., Gullo, L., Daniele, C., Priori, P., Labo', G. (1983) Pancreolauryl test for pancreatic exocrine insufficiency. *Am. J. Gastroent,* **78,** 806–809
12. Fleiss, J.L. (1981) Statistical methods for rates and proportions, *New York: John Wiley and Sons*
13. Bodner, E., Schwamberger, K., Mikuz, G. (1982) Cytological diagnosis of pancreatic tumors. *World J. Surg.*, **6,** 103–106
14. Steinberg, W.M., Gelfand, R., Anderson, K.K. *et al* (1986) Comparison of the sensitivity and specificity of the CA 19-9 and carcinoma embryonic antigen assays in detecting cancer of the pancreas. *Gastroenterology,* **90,** 343–349
15. Beretta, E. *et al* (1987) Serum CA 19-9 in the postsurgical follow-up of patients with pancreatic cancer. *Cancer,* **60,** 2428–2431
16. Kamin, P.D. *et al* (1980) Comparison of Ultrasound and Computed Tomography in the detection of pancreatic malignancy. *Cancer,* **46,** 2410–2412
17. Silverstein, M.D. *et al* (1984) Suspected pancreatic cancer presenting as pain or weight loss: analysis of diagnostic strategies. *World Surg,* **8,** 839–845
18. Enzel, U., Esposti, P.L., Rubio, C., Sigurdson, A., Zajicek, J., (1971) Investigation in tumors spread in connection with aspiration biopsy. *Acta Radiol. (Ther) (Stockh),* **10,** 385–388

### INVITED COMMENTARY

CT-guided fine needle aspiration cytology (FNA) can be extremely helpful in the diagnosis of pancreatic cancer in patients with focal lesions of the pancreas. No false negative results were found by Carlucci and co-workers in 40 patients with pancreatic cancer, proven by surgery or clinical outcome (1). A much lower sensitivity of 68% – 91% has been reported in series of more than 25 patients by others using FNA, guided by CT, Ultrasound (US) or other radiological methods (2). False positive results of FNA are extremely low, reflecting a specificity of 100% found by most authors (2,3). Non-diagnostic tests can occur, in as much of 25%, mainly because of sampling errors (3). These sampling errors had their effect on specificity in the paper of Carlucci et al. (1) but were treated as a separate catagory by others (3).

The ideal diagnostic test for pancreatic cancer should be accurate, non-invasive, without complications and cost-effective. Although CT-guided FNA cytology has some of these characteristics the test is invasive and has complications, such as pancreatitis and seeding of tumor along the needle track (4), although the complications are very infrequent (2). But one individual test can not accurately make the diagnosis in all patients. And a diagnostic strategy, using different tests should be used. A strategy can be chosen by decision analysis weighing accuracy, complication rate and costs of various different strategies as has been clearly demonstrated by Silverstein et al (5). However, the best diagnostic strategy depends also on the prevalence of pancreatic cancer and the presenting symptoms in the group to be studied. Experience of the physicians with the diagnostic, palliative and curative procedures will also play a role in selection of diagnostic strategy.

Pain and jaundice are the two most frequent clinical symptoms of patients with pancreatic and periampullary cancer, frequently accompanyed by weight loss. Resection of pancreatic cancer with hope for cure is practically impossible in non-jaundiced patients, but can be performed in about 80% of the patients with obstructive jaundice in a selected group (6). Furthermore biliary drainage should always be performed in patients with obstructive jaundice, regardless of the chance of cure. Therefore diagnostic approaches will be different for jaundiced and non-jaundiced patients.

In non-jaundiced patients with abdominal pain, suspected of pancreatic cancer, US will be the first step in the diagnostic approach, having a sensitivity of about 70% (5). The sensitivity can be enhanced to 92.9% when positive findings at US are combined with elevated serum levels of the tumor markers CA 19-9 and elastase 1 in small tumors (7). Others find that serum CA 19-9 is of little help in the diagnosis of small pancreatic cancers, and may only be useful in the estimation of tumor load during follow-up (8). Only when US is negative or inadequate a CT-scan is indicated (5). Tumors shown by US or CT should be evaluated using FNA. ERCP should only follow when FNA is negative or non diagnostic. The predictive value of a positive outcome of this strategy is estimated to be more than 99%, regardless of the prevalence of pancreatic cancer but predictive value of a negative test is estimated to be high (99%) when prevalence is low (5%) and relatively low (91%) when prevalence is high (50%). Using this strategy 8.1% of the patients will undergo FNA in the low prevalence group and 45% in the high prevalence group. Diagnostic laparotomy can be as low as 1 – 6% following these principles (5).

In patients suspected of pancreatic cancer with obstructive jaundice as predominant presenting clinical symptom, ERCP is the first step of a diagnostic strategy. US and laboratory tests have usually been performed before to establish the diagnosis of obstructive jaundice. ERCP can have a false positive rate, as low as 5.6%, according to one study (9). An additional advantage of ERCP is that biopsies of the tumor can be obtained in some cases and the degree of tumor invasion and obstruction can be appreciated during endoscopy. Furthermore internal biliary drainage can be achieved by intubation of the tumor with an endoprosthesis (10). In my opinion this should always be done either as a palliative procedure or as a preoperative measure as long as no obstruction of the gastro-intestinal tract is imminent. When curative resection is not taken into consideration because of the poor general condition or advanced age of the patient excellent non-surgical palliation is achieved with acceptable mortality, morbidity and re-intervention rate in very experienced hands (10). For those of us not-experienced in the technique of endoscopic biliary drainage surgery is still the treatment of choice and palliative surgery is also indicated for patients with a relatively long life expectancy, since the chance of serious complications of an endoprosthesis increases over the months. For these patients that are well enough for curative surgery, preoperative internal biliary drainage will improve their condition. Drainage can also have a benificial effect on morbidity and mortality after surgery, probably by leading to a decrease in endotoxinaemia, as has been shown in experimental studies (11,12). It is most likely that the complication rate of internal biliary drainage by the endoscopic route is lower than by the percutaneous transhepatic route (PTD). PTD should be abandoned for this purpose, since in randomized trials the potential beneficial effect of preoperative biliary drainage did not outweighed the complications of this technique (13,14).

At least 2–3 weeks of internal biliary drainage is advised for good results. In that period further work-up should include dynamic contrast-enhanced CT that can predit accurately liver metastases and involvement of local large size veins and arteries (16). Arteriography is thus made superfluous, although I will still prefer it as a "roadmap" for surgery. When distant metastases and local irresectability are diagnosed, FNA cytology of primary tumor or secondaries is useful, especially when non-surgical palliative treatment is considered. But when laparotomy is going to be performed anyhow, tissue diagnosis can be obtained at the time of surgery. The failure rate of preoperative biopsies (16) can probably be improved by the use of intraoperative US (17).

It can be concluded that FNA cytology of suspected pancreatic lesions is an essential step in the diagnostic approach of pancreatic cancer with a high predictive value of a positive test.

By intelligent use of modern diagnostic tests the number of exploratory laparotomies for pancreatic cancer will be very low (5). Despite the high sensitivity of CT-guided FNA reported by Carlucci et al. (1) the high percentage of false negative results in other studies make FNA of limited value in exclusion of cancer with suspected lesions in the pancreas.

H. Obertop
Academische Ziekenhuis Utrecht
Utrecht
The Netherlands

### REFERENCES

1. Carlucci, M., Zerbi, A., Parolini, D., Sironi, S., Vanzulli, A., Staudacher, C., Faravelli A., Garancini, P., Del Maschio, A., Di Carlo, V. CT-guided pancreatic percutaneous fine-needle biopsy in differential diagnosis between pancreatic cancer and chronic pancreatitis.
2. Hall-Craggs, M.A. and Lees, W.R. (1986) Fine-needle aspiration biopsy: pancreatic and biliary tumors. AJR, **147**, 399–403
3. Dickey, J.E., Haaga, J.R., Stellato, T.A., Schultz, C.L., Hau, T. (1986) Evaluation of computed tomography guided percutaneous biopsy of the pancreas. *Surgery, Gynecology & Obstetrics*, **163**, 497–505
4. Cutherell, L., Wanebo, H.J., Tegtmeyer, C.J. (1986) Catheter tract seeding after percutaneous biliary drainage for pancreatic cancer. *Cancer*, **57**, 2057–2060
5. Silverstein, M.D., Richter, J.M., Podolsky, D.K., Warsaw A.L. (1984) Suspected pancreatic cancer presenting as pain or weight loss: analysis of diagnostic strategies. *World Journal of Surgery*, **8**, 839–845
6. Obertop, H., Bruining, H.A., Eeftinck Schattenkerk, M., Eggink, W.F., Jeekel, J., van Houten, H. (1982) Operative approach to cancer of the head of the pancreas and the peri-ampullary region. *British Journal of Surgery*, **69**, 573–576
7. Iishi, H., Yamamura, H., Tatsuta, M, Okuda, S., Kitamura, T. (1986) Value of ultrasonographic examination combined with measurement of serum tumor markers in the diagnosis of pancreatic cancer of less than 3 cm in diameter. *Cancer*, **57**, 1947–1951
8. Sakahara, H., Endo, K., Nakajima, K., Nakashima, T., Koizumi, M., Ohta, H., Hidaka, A., Kohno, S., Nakano, Y., Naito, A., Suzuki, T., Torizuka, K. (1986) Serum CA 19-9 concentrations and computed tomography findings in patients with pancreatic carcinoma. *Cancer*, 57, 1324–1326
9. Gilinsky, N.H., Bornman, P.C., Girdwood, A.H., Marks, I.N. (1986) Diagnostic yield of endoscopic retrograde cholangiopancreatography in carcinoma of the pancreas. *British Journal of Surgery*, **73**, 539–543
10. Huibregtse, K. (1988 Endoscopic biliary and pancreatic drainage. Stuttgart; New York: Thieme pp. 35–42
11. Gouma, D.J., Coelho, J.C.U., Schlegel, J.F., Li, Y.F., Moody, F.G. (1987) The effect of preoperative internal and external biliary drainage on mortality of jaundiced rats. *Arch. Surg.*, **122**, 731–734
12. Gouma, D.J., Coelho, J.C.U., Fisher, J.D., Schlegel, J.F., Li, Y.F., Moody, F.G. (1986) Endotoxaemia after relief on biliary obstruction by internal and external drainage in rats. *Am. J. Surg.*, **151**, 476–479
13. McPherson, G.A.D., Benjamin, I.S., Hodgson, H.F.J., Bowley, N.B., Allison, D.J., Blumgart, L.H. (1984) Preoperative percutaneous transhepatic drainage: the results of a controlled trail. *British Journal of Surgery*, **71**, 371–375
14. Pitt, H.A., Gomes, A.S., Lois, J.F., Mann, L.L., Deutsch, L.S., Longmire, W.P. (1985) Does preoperative percutaneous biliary drainage reduce operative risk or increase hospital costs? *Ann. Surg.*, **201**, 545–553
15. Shimamoto, K., Ishiguchi, T., Sakuma, S. (1987) CT evaluation of pancreatic cancer – analysis of resected tumours. *Eur. J. Radiol.*, **7**, 37–41
16. Campanale, R.P., Frey, C.F., Farias, R., Twomey, P.L., Guernsey, J.M., Keehn, R., Higgins, G. (1985) Reliability and sensitivity of frozen-section pancreatic biopsy. *Arch. Surg.* **120**, 283–288
17. Sigel, B., Coelho, J.C.U., Nyhus, L.M., Velasco, J.M., Donahue, P.E., Wood, D.K., Spigos, D.G. (1982) Detection of pancreatic tumors by ultrasound during surgery. *Arch. Surg.*, **117**, 1058–1061

